# Predicting the Impact of Multiwalled Carbon Nanotubes on the Cement Hydration Products and Durability of Cementitious Matrix Using Artificial Neural Network Modeling Technique

**DOI:** 10.1155/2013/103713

**Published:** 2013-12-30

**Authors:** Babak Fakhim, Abolfazl Hassani, Alimorad Rashidi, Parviz Ghodousi

**Affiliations:** ^1^Civil Engineering and Environment Faculty, Tarbiat Modares University, Tehran 111-14115, Iran; ^2^Nanotechnology Research Center, Research Institute of Petroleum Industry, Tehran, Iran; ^3^Civil Engineering Department, Iran University of Science and Technology, Tehran, Iran

## Abstract

In this study the feasibility of using the artificial neural networks modeling in predicting the effect of MWCNT on amount of cement hydration products and improving the quality of cement hydration products microstructures of cement paste was investigated. To determine the amount of cement hydration products thermogravimetric analysis was used. Two critical parameters of TGA test are PHP_loss_ and CH_loss_. In order to model the TGA test results, the ANN modeling was performed on these parameters separately. In this study, 60% of data are used for model calibration and the remaining 40% are used for model verification. Based on the highest efficiency coefficient and the lowest root mean square error, the best ANN model was chosen. The results of TGA test implied that the cement hydration is enhanced in the presence of the optimum percentage (0.3 wt%) of MWCNT. Moreover, since the efficiency coefficient of the modeling results of CH and PHP loss in both the calibration and verification stages was more than 0.96, it was concluded that the ANN could be used as an accurate tool for modeling the TGA results. Another finding of this study was that the ANN prediction in higher ages was more precise.

## 1. Introduction

Cementitious materials (especially concrete) are the most common construction materials used worldwide. However, cementitious materials are generally brittle and have very low tensile strength and strain capacity. Macroscopic steel reinforcement bars are commonly used to improve the strength and ductility of this type of material, but in recent decades extensive research on the effects of micro- and macrofibers in controlling the growth of cracks in cementitious materials has been carried out [1–6]. In recent times, various nano fibers have raised the interest of researchers due to their exceptional mechanical properties and high potential in reinforcing cement matrix. Carbon nanotube (CNT) is one of the most important areas of research in the field of nanotechnology. CNTs have already proven their reinforcing performance in polymer based materials [1, 2]. In addition to their high strength and elastic constant, CNTs have extremely high aspect ratios, with values typically higher than 1000 : 1 and reaching as high as 2,500,000 : 1 [7]. Carbon nanotube is the most common type of carbon nanostructures. In the early 1990s, Sumio Iijima reported, for the first time, the formation of carbon nanotube. Carbon nanotubes are generally divided into two types, single walled (SWNT) and multiwalled (MWNT). Carbon nanotubes can be represented as a graphene sheet rolled into a cylinder with specific alignment of the hexagonal rings and hemifullerenes attached to the tips [8]. MWNTs can be represented as a family of SWNTs of different diameters, which are combined within a single entity in the form of concentric type MWNTs.

Carbon nanotubes can be considered as an exceptional reinforcing material due to their extremely high aspect ratios [9], ultra high strength [10], modulus [11], and elasticity [12]. The dimensions of nanotubes are at nanoscale which means that they can be distributed within the cement matrix at much more finer scale as compared to traditional reinforcing fibers since reinforcement of cement is typically done at millimeter scale. The application of carbon nanotubes to reinforce cementitious composites is therefore intended to enhance the reinforcing behavior at nanolevel instead of macrolevel. Cracks can be interrupted much more quickly, and the eventually this hinders growth of crack at early stage and prevents propagation of cracks to microscale. In addition, nanotubes have the potential to act as filler within the cement grains, thus producing denser composites. Therefore, CNT reinforcements have the ability to produce significantly stronger and tougher composites as compared to traditional reinforcing fibers. 

In this study, the effect of multiwalled carbon nanotube on cement hydration products especially calcium silicate hydrates (C–S–H) and consequently on the durability of cementitious matrix has been studied. And then, based on the results, the optimum percent of MWCNT has been determined. Finally, based on the TGA analysis, the effect of MWCNT on the amount of cement hydration products and on improving the quality of cement hydration products microstructures of cement paste has been modeled by using artificial neural networks (ANN). Nowadays, thermogravimetric analysis (TGA) is widely accepted and used as a very accurate method in determining the amount of cement hydration products. Recently, some researchers have attempted to model the TGA test results on a variety of composites using artificial neural networks. For example, some researcher in 2010 successfully prepared medium density polyethylene (MDPE) nanocomposite with 3 wt%, 6 wt%, and 9 wt% cloisite Na_, and the thermal stability of nanocomposite was investigated using the thermogravimetric analysis (TGA) [13]. The TGA in air atmosphere showed significantly improved thermal stability of 3 wt%, 6 wt%, and 9 wt% cloisite Na_ nanocomposite in comparison to pure MDPE. The results of TGA of MDPE/cloisite Na_ nanocomposites were predicted by the artificial neural network (ANN). The ANN and adaptive neural fuzzy inference systems (ANFIS) models were developed to predict the degradation of MDPE/cloisite Na_ nanocomposite with temperature. The results revealed that there was a good agreement between predicted thermal behavior and actual values. The findings of the study also showed that the artificial neural networks and ANFIS techniques can be applied as a powerful tool. In another study, a new approach based on artificial neural network (ANN) has been introduced to study the kinetics of thermal decomposition reactions of different polymeric materials, using dynamic thermogravimetry analysis (TGA) at several heating rates. A multilayer neural network model was trained and tested using published experimental data, allowing the proposed model a very good correlation of the weight-loss data. As an example, the same kinetic model had been successfully used at different heating rates, with different polymeric materials such as polyethylene, cellulose and lignin [14].

## 2. Materials and Methodology

**Figure 1:**
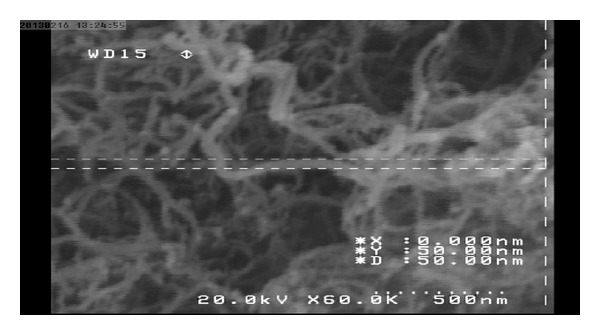
FE-SEM image of the obtained bundles MWCNTs shows a maximum length of 10 *μ*m and diameters of around 50 nm.

**Figure 2:**
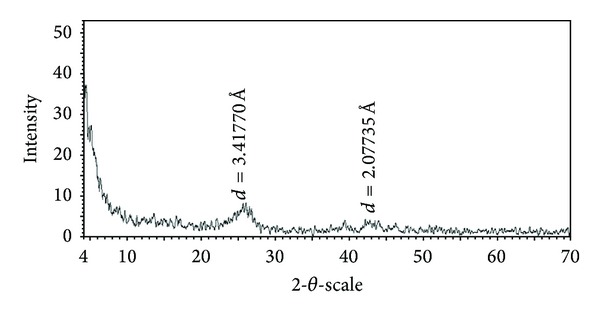
X-ray diffraction patterns of the pristine MWNTs.

Type I Portland cement (Tehran Cement, Iran) was used in this study. Chemical and physical specifications for this type of cement and its allowable ranges in accordance with ASTMC 150 are shown in Tables 1 and 2. In this study, multiwalled carbon nanotubes were used.  Figure 1 shows FE-SEM images of the obtained MWCNTs bundles, which had a length of 10 *μ*m and diameters of around 50 nm.  Figure 2 shows the XRD pattern of the pristine MWNTs. It can be seen that the diagram of pure MWCNTs exhibits the typical peaks at 26.051° and 43.531°, corresponding to the graphite (002) and (100) reflections (Joint Committee for Powder Diffraction Studies (JCPDS) No. 01-0646) [12].

### 2.1. Dispersion of Carbon Nanostructures within Cement Matrix

To disperse the carbon nanotubes within the cement matrix, the MWCNTs was added gradually to water containing polycarboxylate ether (PCE) superplasticizer and the mixture was sonicated for 5 min after each addition to give a total sonication time of 40 min. The sonication conditions were as follows: the amplitude was set to 50%, frequency 20 Hz, power 500 W, titanium alloy probe width 13 mm, and a constant applied energy of 1900 J/min.

After sonication, cement was added to the dispersed MWCNTs at a water/cement ratio of 0.4 and mixed for 30 s using a rotary mixer equipped with a flat beater. This process followed the ASTM C 109 procedure (ASTM C 109/C 109 M, 2008).

### 2.2. Thermogravimetric Analysis (TGA)

The phase changes in hardening cement paste can be monitored by using thermogravimetric analysis (TGA) through measuring the weight of a sample as it is being heated at a controlled rate in a Mettler TGA (model 851 LF). TGA analysis is performed on the cement paste samples containing different percentages (0.1, 0.3, 0.5, and 1.0 wt%) of MWCNTs in comparison with control samples containing no MWCNT with a water/cement ratio of 0.4 at various ages. The ages of testing were 1, 3, 7, 14, 28, and 56 days. The hand mixed pastes were cast into PMMA moulds (18.0 × 5.0 × 5.0 cm), partially filled and sealed on top with polyethylene films. The moulds were vibrated for one minute to remove any air bubbles and voids. After 1 day, the hardened blocks (18.0 × 5.0 × 5.0 cm) were demoulded, crushed into smaller pieces, put in the moulds, and sealed again. The environmental chamber, where the samples were cured, was at 23 ± 1°C and 50% RH. At different curing times crushed samples of each series were extracted from the ageing chamber and dried to constant weight at 85°C for 8 h, to stop hydration: free liquid was thereby eliminated. Dried samples were grounded in an agate mortar and sieved to minus 80 mesh and the resulting powders were stored in desiccators. In this study, TGA measures the weight loss of a powdered sample which is subjected to heating from 25°C to 1000°C at a heating rate of the 10°C/min in flowing argon (Ar) atmosphere.

To some extent various hydrate phases decompose (liberate their water) at different temperature ranges. Though these ranges overlap considerably, some distinctions can be made between calcium hydroxide (CH) and other hydrates which include the primary product of hydration, calcium silicate hydrate (C–S–H). Thermogravimetric analysis has been widely accepted as an accurate method for the determination of crystalline calcium hydroxide (CH) content [15]. Calcium hydroxide is mostly crystalline and nonporous, and it decomposes between about 400 and 450°C. Weight loss between 400 and 450°C will be referred to as the “calcium hydroxide loss (CH_loss_).” Though only weight loss from the decomposition of CH is measured, it is shown that this weight loss is very close to the amount of water in CH and therefore proportional to the amount of CH [15]. The presence of mass loss between 105°C and 400°C includes the loss of water associated with the amorphous and porous hydration products, the majority of which is the C–S–H gel [16]. Weight loss computed over this temperature range will thus be referred to as the “porous hydration products loss (PHP_loss_).” Since C–S–H is very difficult to measure directly due to both a lack of crystallinity and an indefinite composition, the weight loss between 105°C and 400°C can be treated due to the decomposition of C–S–H.

Meanwhile, it is worth mentioning that the thermogravimetric analysis also showed that functionalized MWCNTs decompose between about 500°C and 600°C. But since the amount of MWCNTs in this cement matrix was so much little, the TGA curve of cement matrix reinforced with multiwalled carbon nanotubes was so similar to the typical TGA curve of the cement matrix.

**Figure 3:**
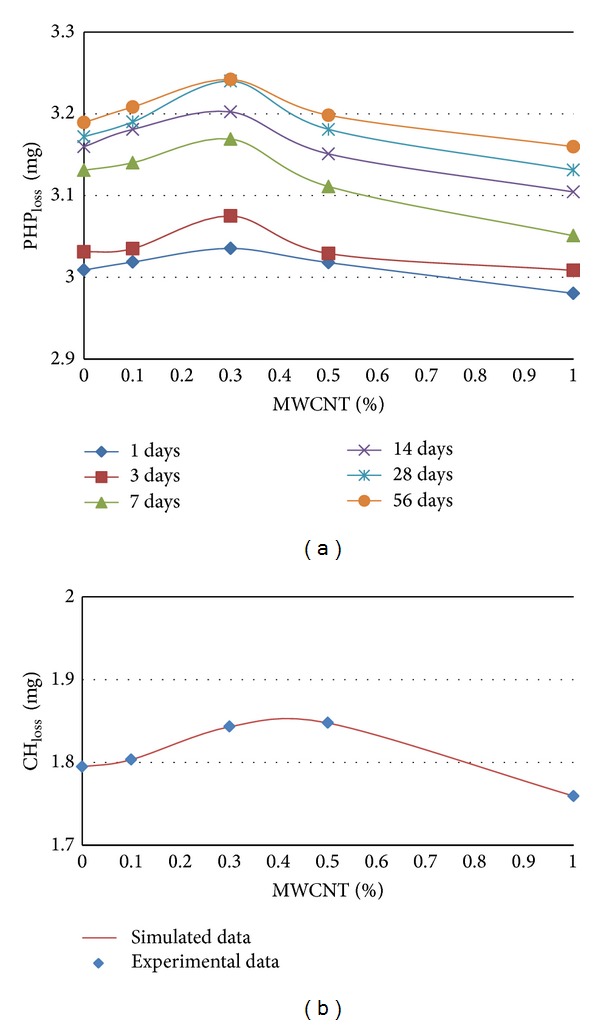
TGA results over time for paste cement with MWCNT at w/cm = 0.4, the calculated PHP_loss_ (a) and CH_loss_ (b) over time due to the evolution of hydration for different cement paste samples containing different percentages (0.1, 0.3, 0.5, and 1.0 wt%) of MWCNTs in comparison with control samples containing no MWCNT with w/cm = 0.4.

The calculated PHP loss and CH loss over time due to the evolution of hydration are plotted in Figure 3 for different cement paste samples containing different percentages (0.1, 0.3, 0.5, and 1.0 wt%) of MWCNTs in comparison with control samples containing no MWCNT with w/cm = 0.4.

As shown in Figure 3 the cement hydration is enhanced in the presence of the MWCNT. As can be seen from Figure 3(a), PHP content (indicated by PHP loss) in cement paste samples containing 0.1, 0.3, 0.5 wt% exceeds the control cement paste during the hydration, indicating that nucleation of C–S–H on the MWCNT accelerated the dissolution, nucleation, and growth of the hydration products compared with the normal cement mortar. C–S–H or PHP is responsible for the development of many mechanical and physical properties such as strength, permeability, and shrinkage and consequently for improving durability [17].

Increase in MWCNTs while the water/cement ratio of matrix is held constant, causes difficulty in providing suitable workability and consequently dispersing MWCNT within the matrix due to the presence of hydrophilic groups on the MWCNT surfaces. In this circumstance, MWCNTs absorb a nonnegligible amount of water, hampering the hydration of the cement mortar and also causing them to agglomerate in the form of clumps which are very difficult to disentangle. These agglomerates form large voids within the cement matrix and stresses cannot be transferred across the bundles. In addition, if the MWCNT bundles remain intact, they no longer remain within the nanoscale range. Instead of filling the nanosized void spaces within the cement grains, they gather between cement hydration products and create zones of weakness throughout the cement matrix. This could be why PHP content (indicated by PHP_loss_) and CH content (indicated by CH_loss_) in cement paste samples containing 1.0 wt% are much less than that of the control samples (Figure 3).

### 2.3. Artificial Neural Networks

Artificial neural networks (ANNs) are data processing systems consisting of a large number of simple, highly interconnected processing elements (artificial neurons) in an architecture inspired by the structure of the central cortex of the brain [18, 19]. Much of the success of neural networks is due to such characteristics as nonlinear processing and parallel processing. Neural network modeling techniques have been rapidly applied in engineering, business, psychology, science, and medicine in recent years. In civil engineering, the methodology of neural networks has been successfully applied to a number of areas such as structural analysis and design [20, 21], structural damage assessment [22, 23], structural dynamics and control [24], seismic liquefaction prediction [25], constitutive modeling [26–28], pavement condition-rating modeling [29], and evaluating CPT calibration chamber test data [30].

**Figure 4:**
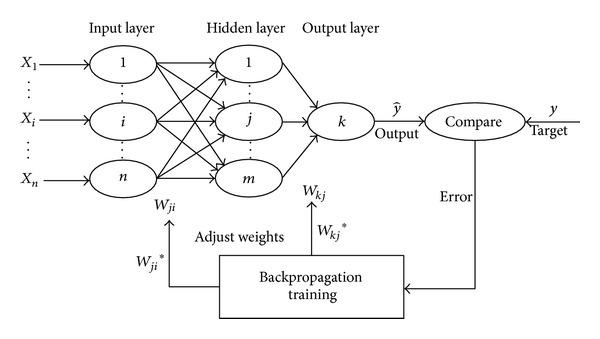
A three-layered FFNN with a backpropagation training algorithm.

In this study for ANN modeling, the multilayer perceptron (MLP) is used. A multilayer perceptron (MLP) is a feed forward artificial neural network model. An MLP consists of multiple layers of nodes in a directed graph, with each layer fully connected to the next one. Three-layered feed forward neural network (FFNN) shown in Figure 4 is used in this study. This network is usually applied in forecasting time series and in providing a general framework for representing nonlinear functional mapping between a set of input and output variables [31].

Three-layered FFNNs are based on a linear combination of the input variables, which are transformed by a nonlinear activation function. The explicit expression for an output value of FFNNs is given by following equation:

(1) $$\begin{array}{c} {\hat{y}}_{k}=f_{o}\left[\overset{m}{\underset{j=1}{\sum }}w_{kj}\cdot f_{h}\left(\overset{n}{\underset{i=1}{\sum }}w_{ji}x_{i}+w_{jo}\right)+w_{ko}\right], \end{array}$$

where *w*_*ji*_ is the weight in the hidden layer connecting the *i*th neuron in the input layer and the *j*th neuron in the hidden layer; *w*_*jo*_ is the bias for the *j*th hidden neuron; *f*_*h*_ is the activation function of the hidden neuron; *w*_*kj*_ is the weight in the output layer connecting the *j*th neuron in the hidden layer and the *k*th neuron in the output layer; *w*_*ko*_ is the bias for the *k*th output neuron; *f*_*o*_ is the activation function for the output neuron. The weights are different in the hidden and output layer, and their values can be changed during the process of network training.

### 2.4. Goodness-of-Fit Tests

To examine how close the predicted data to the experimental ones, some different criteria are used. There are two types of graphical and statistical criteria goodness of fit where each has its own unique features, and is used for a specific purpose. Since the graphical method is not accurate and varies depending on opinion of individual person, the statistical criteria were used in this study. Efficiency coefficient (EC) and the root mean square error (RMSE) are the most widely used statistical criteria [32, 33]. Finally, the best model, based on the highest efficiency and the lowest root mean square error coefficient, is chosen:

(2) $$\begin{array}{c} \text{EC}=1-\frac{\sum_{i=1}^{n}{\left(X_{ei}-X_{si}\right)}^{2}}{\sum_{i=1}^{n}{\left(X_{ei}-\bar{X_{e}}\right)}^{2}},\;-\infty <\text{EC}\leq 1,\\ \text{RMSE}={\left[\frac{1}{n}\overset{n}{\underset{i=1}{\sum }}{\left(X_{oi}-X_{ci}\right)}^{2}\right]}^{1/2}, \end{array}$$

where *n* is the number of data, *X*_*si*_ is experimental data, *X*_*ei*_ is simulated data, and $\overset{-}{X_{e}}$ is average of experimental data.

## 3. Results and Discussions

### 3.1. Neural Networks on TGA Test Results

As mentioned above, two main and critical parameters of TGA test are PHP_loss_ and CH_loss_. So in order to model the TGA test results, the ANN modeling must be performed on these parameters separately.

In this study, 60% of data are used for model calibration and the remaining 40% are used for model verification. Accordingly, the data are normalized according to the following formula and is then used in the neural networks [34]

(3) $$\begin{array}{c} Y_{i}=\frac{X_{si}}{X_{s\max}},\;i=1,2,3,\ldots ,n, \end{array}$$

where *Y*_*i*_ is the normalized data, *X*_*si*_ is the input data, and *X*_*s*max⁡_ is the maximum of the input data.

The process of network training is accomplished by a feedback propagation algorithm. This algorithm is based on the error-correction learning rule of Levenberg-Marquardt (LM). The activation function is hyperbolic tangent sigmoid (TANSIG) type. This neural network model has one input layer, one output layer, and one hidden layer. The modeling process is performed by the ANN toolbox in the MATLAB environment.

### 3.2. Modeling of PHP_**loss**_ by Artificial Neural Network

In order to predict the PHP_loss_ values for 7, 14, 28, and 56 ages, (4) to (7) were used, respectively:

(4) $$\begin{array}{c} {\text{PHP}}_{\text{TGA}7\text{D}}=f\left({\text{PHP}}_{\text{TGA}3\text{D}},{\text{PHP}}_{\text{TGA}1\text{D}}\right), \end{array}$$

(5) $$\begin{array}{c} {\text{PHP}}_{\text{TGA}14\text{D}}=f\left({\text{PHP}}_{\text{TGA}7\text{D}},{\text{PHP}}_{\text{TGA}3\text{D}},{\text{PHP}}_{\text{TGA}1\text{D}}\right), \end{array}$$

(6) PHPTGA28D=f(PHPTGA14D,PHPTGA7D,  PHPTGA3D,PHPTGA1D),

(7) PHPTGA56D=f(PHPTGA28D,PHPTGA14D,PHPTGA7D,  PHPTGA3D,PHPTGA1D).

The goodness-of-fit test results (RMSE and EC) of PHP_loss_ modeling using artificial neural networks are presented in Table 3.

**Figure 5:**
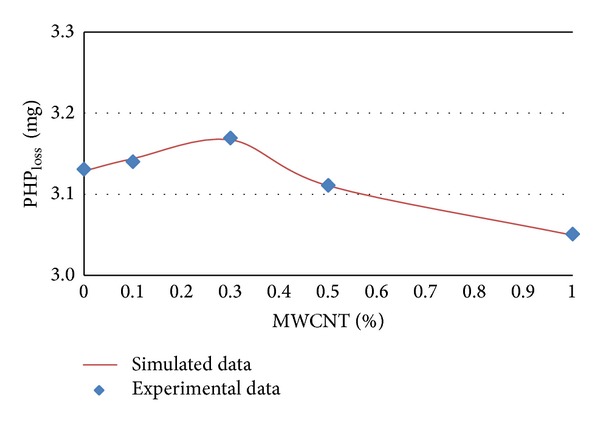
Experimental and simulated values of PHP_loss_ for 7 days by artificial neural networks.

The experimental and simulated data of PHP_loss_ in terms of MWCNTs content percentages for different ages of 7, 14, 28, and 56 days are shown in Figures 5 and 8, respectively.

**Figure 6:**
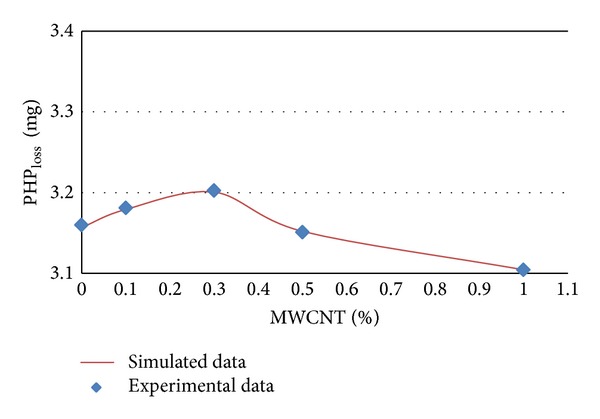
Experimental and simulated values of PHP_loss_ for 14 days by artificial neural networks.

**Figure 7:**
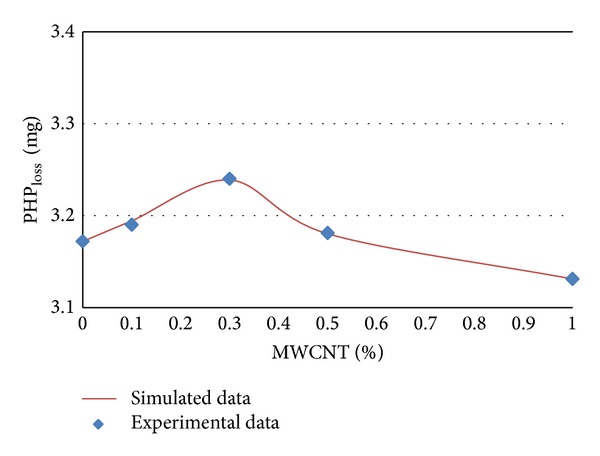
Experimental and simulated values of PHP_loss_  for 28 days by artificial neural networks.

**Figure 8:**
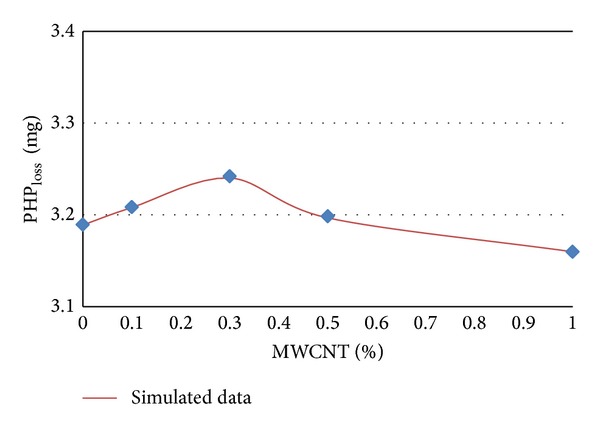
Experimental and simulated values of PHP_loss_ for 56 days by artificial neural networks.

It can be concluded from Table 3 and Figures 4, 5, 6, and 7 that PHP_loss_ values can be efficiently modeled by artificial neural network at ages of 7, 14, 28, and 56 days. Furthermore, it was shown that the prediction of PHP_loss_ in higher ages was more accurate. It is because of increasing the number of input variables of ANN method in higher ages samples.

### 3.3. Modeling of CH_**loss**_ by Artificial Neural Network

In order to predict the CH_loss_ values for 7, 14, 28, and 56 ages, (8) to (11) were used, respectively:

(8) $$\begin{array}{c} {\text{CH}}_{\text{TGA}7\text{D}}=f\left({\text{CH}}_{\text{TGA}3\text{D}},{\text{CH}}_{\text{TGA}1\text{D}}\right), \end{array}$$

(9) $$\begin{array}{c} {\text{CH}}_{\text{TGA}14\text{D}}=f\left({\text{CH}}_{\text{TGA}7\text{D}},{\text{CH}}_{\text{TGA}3\text{D}},{\text{CH}}_{\text{TGA}1\text{D}}\right), \end{array}$$

(10) $$\begin{array}{c} {\text{CH}}_{\text{TGA}28\text{D}}=f\left({\text{CH}}_{\text{TGA}14\text{D}},{\text{CH}}_{\text{TGA}7\text{D}},{\text{CH}}_{\text{TGA}3\text{D}},{\text{CH}}_{\text{TGA}1\text{D}}\right), \end{array}$$

(11) CHTGA56D=f(CHTGA28D,CHTGA14D,CHTGA7D, CHTGA3D,CHTGA1D).

The goodness-of-fit test results (RMSE and EC) of CH_loss_ modeling using artificial neural networks are presented in Table 4.

**Figure 9:**
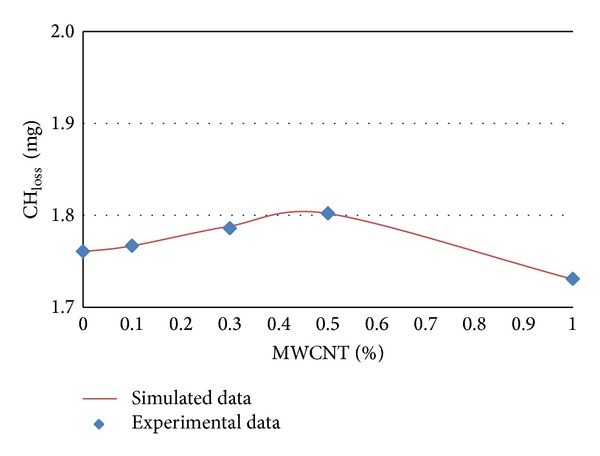
Experimental and simulated values of CH_loss_ for 7 days by artificial neural networks.

The experimental and simulated data of CH_loss_ in terms of MWCNTs content percentages for different ages of 7, 14, 28, and 56 days are shown in Figures 9 and 12, respectively.

**Figure 10:**
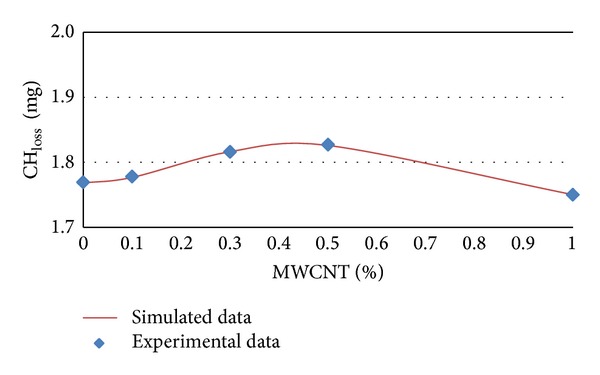
Experimental and simulated values of CH_loss_ for 14 days by artificial neural networks.

**Figure 11:**
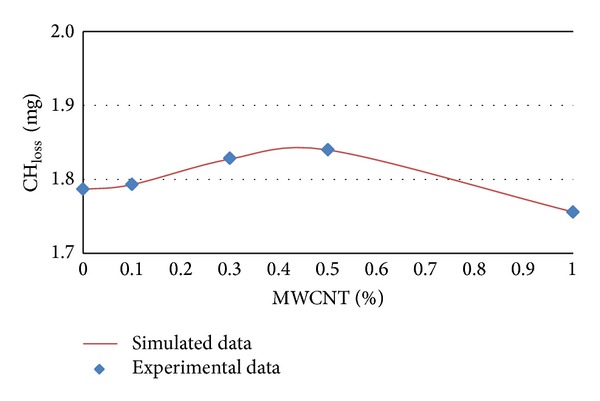
Experimental and simulated values of CH_loss_ for 28 days by artificial neural networks.

**Figure 12:**
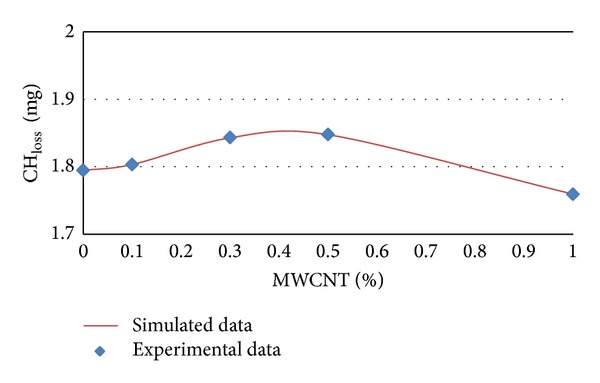
Experimental and simulated values of CH_loss_ for 56 days by artificial neural networks.

It can be concluded from Table 4 and Figures 9, 10, 11, and 12 that CH_loss_ values can be efficiently modeled by artificial neural network at ages of 7, 14, 28, and 56 days. Furthermore, similar to the PHP_loss_ modeling, the prediction of CH_loss_ in higher ages because of increasing the number of input variables was more precise.

## 4. Conclusions

1. The results of TGA test implied that the cement hydration is enhanced in the presence of the optimum percentage of MWCNT. It can be seen that PHP content (indicated by PHP loss) in cement paste samples containing 0.1, 0.3, and 0.5 wt% exceeds the control cement paste during the hydration. Therefore, due to the point that the amount of C–S–H, the most desirable cement hydration products, in the presence of the 3% of MWCNTs was the highest, it can be concluded that the optimum content of MWCNT is 0.3 wt%.
2. Increase in MWCNTs while the water/cement ratio of matrix is held constant, due to the presence of hydrophilic groups on the MWCNT surfaces and consequently absorption of a nonnegligible amount of water, caused hampering of the hydration of the cement mortar and agglomerating MWCNTs in the form of clumps. Therefore, hampering the hydration of the cement mortar is the main reason of the significant decrease in CH and PHP loss sample values containing 1.0% MWCNTs compared to the other samples.
3. Since the efficiency coefficient (EC) of the modeling results of CH and PHP loss using artificial neural networks in both the calibration and verification stages is more than 0.96, it can be concluded that the ANN can be used as an accurate and ultrafast tool for modeling the TGA test results.
4. This study also showed that the PHP and CH loss values of samples can be easily ANN-modeled by using those values for smaller age samples. For instances, by using the CH and PHP loss values of 1, 3, 7, 14, and 28 days samples, the CH_loss_ and PHP_loss_ of 56 days samples can be ANN-modeled, easily.
5. Another finding of this study was that the ANN prediction of CH_loss_ and PHP_loss_ in higher ages because of increasing the number of input variables and consequently more concentration on input data to achieve optimum result was more precise.

## Figures and Tables

**Table 1:** Chemical contents of Type I cement, according to ASTM C 150.

Constituent compounds	CaO, %	SiO_2_, %	Al_2_O_3_, %	Fe_2_O_3_, %	MgO, %	SO_3_, %	L.O.I, %	I.R, %

Measured value	62.35	21.45	4.61	3.3	3.26	2.05	2.00	0.57

**Table 2:** Properties of Type I cement, according to ASTM C 150.

	Chemical properties			Physical properties						
	MgO	L.O.I	I.R	Blaine specific surface cm^2^/g	Autoclave expansion, %	Setting time		Compressive strength kg/cm^2^		
	%	%	%			Initial minutes	Final hours	2-days	At least 28 days	At last 28 days
Value	<5	<3	<0.75	>2800	<0.8	>45	<6	>100	>425	<625

**Table 3:** The results of PHP_loss_ modeling method based on artificial neural networks.

RMSE		EC		Network model	Type
Verification phase	Calibration phase	Verification phase	Calibration phase		
0.0031	0.0015	0.9557	0.9981	2-3-1	PHP_loss_ 7 day
0.0018	0.0018	0.9724	0.9942	3-3-1	PHP_loss_ 14 day
0.0031	0.0004	0.9842	0.9996	4-4-1	PHP_loss_ 28 day
0.0013	0.0010	0.9937	0.9966	5-4-1	PHP_loss_ 56 day

**Table 4:** The results of PHP_loss_ modeling method based on artificial neural networks.

RMSE		EC		Network model	Type
Verification phase	Calibration phase	Verification phase	Calibration phase		
0.0018	0.0003	0.9511	0.9961	2-3-1	PHP_loss_ 7 day
0.0008	0.0003	0.9791	0.9955	3-3-1	PHP_loss_ 14 day
0.0007	0.0002	0.9838	0.9998	4-4-1	PHP_loss_ 28 day
0.0002	0.0001	0.9950	0.9999	5-4-1	PHP_loss_ 56 day
